# A randomised controlled trial to test the effectiveness of decision training on assessors’ ability to determine optimal fitness-to-drive recommendations for older or disabled drivers

**DOI:** 10.1186/s12909-018-1131-4

**Published:** 2018-02-13

**Authors:** Priscilla Harries, Carolyn Unsworth, Hulya Gokalp, Miranda Davies, Christopher Tomlinson, Luke Harries

**Affiliations:** 1 Brunel University London, London, UK; 2 Central Queensland University, Melbourne, Australia; 3 Curtin University, Perth, Australia; 4 Jonkoping University, Jonkoping, Sweden; 5 Brunel University London, London, UK; 6 Nuffield Trust, London, UK; 7 Imperial College London, London, UK; 8 University of Cambridge, Cambridge, UK

**Keywords:** Decision making, Occupational therapy, Automobile driving, Training

## Abstract

**Background:**

Driving licensing jurisdictions require detailed assessments of fitness-to-drive from occupational therapy driver assessors (OTDAs). We developed decision training based on the recommendations of expert OTDAs, to enhance novices’ capacity to make optimal fitness-to-drive decisions. The aim of this research was to determine effectiveness of training on novice occupational therapists’ ability to make fitness-to-drive decisions.

**Methods:**

A double blind, parallel, randomised controlled trial was conducted to test the effectiveness of decision training on novices’ fitness-to-drive recommendations. Both groups made recommendations on a series of 64 case scenarios with the intervention group receiving training after reviewing two thirds of the cases; the control group, at this same point, just received a message of encouragement to continue. Participants were occupational therapy students on UK and Australian pre-registration programmes who individually took part online, following the website instructions. The main outcome of training was the reduction in mean difference between novice and expert recommendations on the cases.

**Results:**

Two hundred eighty-nine novices were randomised into intervention; 166 completed the trial (70 in intervention; 96 in control). No statistical differences in scores were found pre-training. Post training, the control group showed no significant change in recommendations compared to the experts (t(96) = −.69; *p* = .5), whereas the intervention group exhibited a significant change (t(69) = 6.89; *p* < 0.001). For the intervention group, the mean difference compared with the experts’ recommendations reduced with 95% CI from −.13 to .09. Effect size calculated at the post-training demonstrated a moderate effect (d = .69, *r* = .32).

**Conclusions:**

Novices who received the decision training were able to change their recommendations whereas those who did not receive training did not. Those receiving training became more able to identify drivers who were not fit-to-drive, as measured against experts’ decisions on the same cases.

This research demonstrated that novice occupational therapists can be trained to make decisions more aligned to those of expert OTDAs. The decision training and cases have been launched as a free training resource at www.fitnesstodrive.com. This can be used by novice driver assessors to increase their skill to identify drivers who are, and are not fit-to-drive, potentially increasing international workforce capacity in this growing field of practice.

**Electronic supplementary material:**

The online version of this article (10.1186/s12909-018-1131-4) contains supplementary material, which is available to authorized users.

## Background

Driving is a valued aspect of community mobility; it enables occupational engagement and community participation, and enhances quality of life [1]. However, health conditions and disability can affect driving capacity. Driver assessment, to determine fitness-to-drive, may be undertaken by driver assessors employed by licensing authorities, medical personnel or Occupational Therapist Driving Assessors (OTDAs). OTDAs, with their health-performance expertise, are well placed to conduct such assessments as they are trained in the relationship between health conditions and occupational performance capacity. Internationally, OTDAs assess those with disabilities who would like to learn to drive, as well as those drivers with illness, sudden onset disability, deteriorating health conditions or progressive disabilities. People with age related disabilities (frailty), neurological disorders (e.g. multiple sclerosis), cardiovascular disorder (e.g. stroke), muscular skeletal disorders (e.g. amputation), mental health needs (depression), or disorders with fluctuating disabilities such as dementia, may all require an OTDA driving assessment to determine safety to drive.

Government policy in many countries recognises OTDA’s role as driver rehabilitation specialists in driving assessment. For example the American Medical Association’s Physician’s Guide to Assessing and Counseling older drivers [2], states that a physician may advise a patient to “…*consult a driver rehabilitation specialist. This type of instructor, typically an occupational therapist will go out on the road with you to watch you drive, then recommend ways to make your driving safer.”* In the UK, the Quality, Innovation, Productivity and Prevention Allied Healthcare Professionals Stroke toolkit [3] states that occupational therapists “should assess and redevelop any impairments in skills….and advise patients on the consequences for driving” and where capacity is in doubt, use on-road assessments to assess driving ability [4]. In the Australian State of Victoria, OTDAs have been legally authorised to make recommendations to the licensing authorities regarding fitness-to-drive since 1986.

OTDAs can provide clinic based and on road driver rehabilitation services where required, however, there is a lack of OTDAs internationally and therefore limited services in this area of growing population need. For example there are less than 40 experienced UK OTDAs in this field (UK population of 62 million), as compared with 400+ Australian OTDA: Australian population 22 million. The number of younger disabled drivers who will seek to improve their quality of life through increased mobility, coupled with increasing numbers of older drivers means the number of driver assessments undertaken is expected to rise dramatically over the coming decades [5, 6]. Accurate assessment of fitness-to-drive is necessary not just for increasing road safety but also for avoiding major implications of license cancellation on a person’s life style and potential increased need for family and community support [7].

Over thirty years of research has been undertaken to try to identify the most effective approach to driver assessment. While systematic reviews have identified a range of off-road assessments that can be used to assist determine fitness to drive, the sensitively (correctly identifying drivers who cannot drive) and specificity (correctly identifying drivers who can drive) values of many of these assessments are inadequate [8, 9]. Furthermore, although attempts to present standard protocols for on-road testing have been made [10], the development of a standardised on-road assessment is generally viewed as unattainable due to the fact that each driving situation presents very differently [6]. Instead, researchers have begun to document that there is both the health professional’s judgment of the driver’s capacity to drive, as well as the ‘truth’ of their driving capacity. This means that while optimal recommendations can be made (correctly identifying drivers who can and cannot drive), sub optimal recommendations can also be made (identifying fit drivers as unfit, and the reverse) [11]. Therefore, experts’ clinical judgement is key; experts have to make recommendations based on the findings of both clinic based and on road assessments. The accuracy of these recommendations is essential: they need to prevent unsafe drivers from driving in order to protect the driver and enhance safety for other road users, while not preventing clients who are fit-to-drive from driving.

If we are to look to expert OTDAs for guidance, we need to be aware that inconsistent rulings can be seen in driver assessment decisions made to licensing authorities by driver assessor occupational therapists. For example, in a recent audit undertaken by one of seven licensing authorities in Australia with 46 occupational therapists, it was found that in 25% of cases, the rationale for the recommendation was unclear, and in some cases where instructor intervention was required (which is documented in Competency Standards as automatic fail decisions), 10% of OTDAs were still passing clients [12]. However, we also know it is possible to statistically model how decisions are made, identify the optimal judgement policies that produce these decisions and to use these to improve decision making capacity [13].

A research programme was therefore developed to model how experts make fitness-to-drive recommendations, to identify optimal judgement policies that produce these decisions, and use these policies to enhance decision making capacity of novices. The program comprised two major phases: i) optimal policy capturing study by using a Social Judgement Theory (SJT) approach and development of training materials for a web-based decision training, and ii) a Randomised Controlled Trial (RCT) to test the effectiveness of the web-based decision training tool. The first phase has been published [14], and a short summary is provided.

Initially, we used a qualitative approach to establish the decision context, types of information (cues) available to the decision maker and possible decision outcomes that could be selected. From Unsworth’s previous study [15], the key cues important for making fitness-to-drive recommendations had been identified; these were debated with international OTDAs and adapted in terms of terminology and relevance for international use. Twelve cues were identified as important when making fitness-to-drive recommendations: driving instructor intervention, driver behaviour, cognitive and perceptual skills, vehicle handling skills, road law/road craft knowledge, physical skills, sensory functions, medical prognosis, current driving needs, driving experience and history, residence and age. A written definition was produced for the final 12 cues, and three distinct levels of each cue were established to represent a range of case presentations, with level three being the most positive, representing the lowest impact on fitness-to-drive (see Additional file 1) e.g. the three levels for the driving history cue were 1= ‘Client has had a major accident in the last 12 months’, 2= ‘Client has had a few minor scrapes in the last 12 months’, 3= ‘Client has had no accidents in the last 12 months’. The range and scope of decision recommendations in current practice were identified and agreed. Valid ‘driving’ case scenarios (*n* = 64) of people with disabilities and/or older people were created using fractional factorial design in SPSS. The inter-cue correlations between the 12 cues across the original 64 scenarios were below 0.2. The case scenarios were reviewed, debated and agreed by the project advisory board, the project expert panel and the service user group. An example of a case scenario is given in Additional file 2. Once agreed, the case scenarios along with instructions were put onto a dedicated website ready for assessment by experienced OTDAs in the optimal policy capturing study. There were four possible fitness-to-drive recommendations identified as needed for each case referral:

1. Not fit-to-drive – Suspend or cancel licence
2. Not fit-to-drive – Driver rehabilitation to be completed
3. Fit-to-drive – With conditions, for example, using an automatic car
4. Fit-to-drive – Unrestricted licence

Forty five experienced OTDAs from the UK, Australia and New Zealand made fitness-to-drive recommendations for a set of 64 case scenarios via the dedicated website. Agreement (consensus) between experts’ fitness-to-drive recommendations was very high: intra-class correlation (ICC) ICC(2,1) = .97, 95% confidence interval (.96–.98); the experts also showed excellent consistency on repeated cases ICC(1,1) = .98, 95% confidence interval (.96–.99). Decisions made by the experienced OTDAs were analysed and mathematically modelled using the SJT approach. Two types of analysis were undertaken: Multiple Regression Analysis and Discriminate Function Analysis. The Multiple Regression Analysis, where the dependent variable was the mean expert decisions for original scenarios and the predictor variables were the cue levels for these scenarios, revealed that seven of the 12 cues had a significant influence on fitness-to-drive decisions. These were, in descending order of importance: driving instructor intervention, client’s vehicle handling skills, road law knowledge, physical skills, sensory functions, cognitive and perceptual skills, and driving behaviour. The Discriminate Function Analysis was then used to identify which cues were key to sub groupings (functions) of decision outcomes; this showed that five cues were key to the most important subgrouping i.e. fit-to-drive versus not fit-to-drive. An expert panel and project advisory board reviewed and debated results, finally agreeing on an optimal consensus judgement policy for use in training. Training materials were then developed to train OTDAs to make optimal fitness-to-drive recommendations, and these can be viewed on the training website www.fitnesstodrive.com.

This paper reports on the second phase of the research program. The aim was to test the effectiveness of the training package on novices’ fitness-to-drive recommendations, using a randomised controlled trial. The novices were pre-registration occupational therapy students. Occupational therapy students learn how to analyse activities of daily living as part of their pre-registration education; the depth of understanding is only at a basic competence level until post registration experience has been gained. In some countries, there may be a formal fitness to drive training programme available to be undertaken after working in the field, but this is still a rare opportunity. Therefore pre-registration students, as they are required to have basic knowledge in this field of practice, were suitable novices to train. It was important to determine if the experimental group of novices could make fitness-to-drive recommendations that more closely aligned to expert OTDAs recommendations following training, when compared with recommendations made by control group novices.

## Methods

### Study design

A Randomised Controlled Trial (RCT) was conducted to test the effectiveness of the web-based decision training tool on novice occupational therapists. The study design for the RCT was a parallel, double blind, two-factor mixed design with one between-subjects factor (group) with two levels (control and intervention groups) and one within-subjects factor (time-point or training) with two levels (pre-training and post-training).

### Case scenarios and training materials

The same 64 driving case scenarios used in the first Phase of the research, i.e. the optimal policy capturing study with the experts, were used in the RCT. These contained the 12 cues (independent variables), each with three levels used to create a range of driver skill level (see Additional file 1). Of the 64 case scenarios, 47 were presented at pre-training and 17 at post-training. The number of scenarios used for pre-training and post-training used with the intervention group were calculated using the ratio calculations for judgment analysis [16]. An example of a case scenario and the four possible fitness-to-drive recommendations are presented in Additional file 2. The training materials used in the decision training intervention arm were developed using the findings of the optimal policy capturing study and provided a short description of background to the research and information to help the novices develop their capacity to make fitness-to-drive recommendations. The materials then included detailed information about how the experts used cue levels of the most important cues when making fitness-to-drive recommendations. Overall, the more each cue suggested the client was a safe driver (for instance, if it was reported that the client’s vehicle handling skills supported safe driving), the more likely the OTDA would be to make a judgement of ‘fit-to-drive – unrestricted licence’. In instances where the cue content raised concerns about the client’s ability to drive safely (for instance, where it was reported that physical skills did not support safe driving - no vehicle modifications / compensatory strategies suitable), it was then more likely that the OTDA would recommend ‘not fit-to-drive - suspend or cancel licence’.

### Sample for the RCT study

The participants were occupational therapy students on pre-registration programmes (referred as ‘novices’ herein). As there are insufficient numbers of novice OTDAs at any one time in any one location, it was determined that using pre-registration students would serve the same purpose. It was reasoned that if the training program was effective with pre-registration students, it would also be effective with occupational therapists training to be OTDAs. A sample size calculation identified that 150 novice (75 in each group) were needed to be recruited to identify a medium effect (*r* = .3) of impact of the training intervention on novice decision making [17]. The participants were recruited from across the UK and Australia via Universities; they were invited to participate via programme leads who forwarded them an invitation email. This contained the link to the research website where they could read the information sheet and indicate if they would like to participate. If they clicked on the request to participate they were randomised into the study. Participants were randomised into control and intervention groups by the computer equivalent of tossing a coin when they registered to take part in the experiment. 289 pre-registration students were randomised into the study. Data collection occurred over a three month period.

### Procedure

Ethical Approval for the study was obtained from Brunel University Research Ethics Committee (13/10/STF/02) and from La Trobe University Human Ethics Committee (HEC12–105). The trial was not required to be registered as it was not a clinical intervention trial; it did not involve any impact on patients’ care or clinicians’ practice as it did not use health-related interventions to evaluate the effects on health outcomes. The study evaluated the impact of an educational intervention on pre-registration students’ theoretical recommendations on fitness-to -drive only.

Participants were informed of the study but blinded to the randomisation element. To maintain blinding of investigators, roles were carefully organised. Participants were automatically randomised into one of two conditions, either intervention, or control by the web site using coin toss approach. HG without awareness of respondents’ allocations undertook the analysis. Only after analysis was complete were the allocations revealed.

Following randomisation, participants were e-mailed a password to log-on the website where some demographic information was collected. They were then presented with instructions and two practice scenarios. Participants were presented with the same set of scenarios that were used in Phase 1 of the research (the optimal policy capturing study during which the experts OTDAs made their recommendations). The set of scenarios comprised of 47 pre-training scenarios and 17 post-training scenarios. The scenarios in each set were presented one at a time and in a randomised order to each participant. Participants were asked to choose their recommendation for fitness-to-drive by clicking one of the four fitness-to-drive recommendations as noted above. When the scenarios in the pre-training phase were completed, the intervention group was provided with the training information, whereas the control group was told how many scenarios they had completed and to continue on to complete the whole task. On completion, participants were sent a £10/$15 honorarium gift voucher.

### Data analysis

Each case scenario had four possible fitness-to-drive recommendations which were coded as follows: 1= “Not fit-to-drive: Suspend or cancel licence”; 2= “Not fit-to-drive - driver rehabilitation to be completed (may require reassessment)”; 3=“Fit-to-drive: with conditions such as using an automatic car” and 4=“Fit-to-drive: Unrestricted licence”. The data were treated parametrically [18]. Decisions made by novice participants were compared with the expert consensus recommendation, which was estimated as the mean of rates/decisions given by the experts for that scenario. The results from the control and intervention groups for pre- and post-training were then pooled, giving four sets of results. We compared the results across the groups at pre-training to determine if the two groups possessed similar levels of skill and then at post-training to examine the effect of the training as compared to experts.

Two methods were used to assess the effectiveness of the training intervention: Bland-Altman test [19–21], and Signal Detection Theory (SDT) [22–24]. The Bland-Altman test is based on analysis of differences between recommendations made by each novice and the expert consensus recommendations, and can provide a measure of agreement between each fitness-to-drive recommendations made by the individual novice and the expert consensus recommendation for the same case. Using the Bland-Altman test, for each case scenario, we subtracted the expert consensus recommendation (i.e. ranging from 1 to 4) from the recommendation made by the novice. We then calculated the mean of differences (referred to as the Bland-Altman statistic herein) for pre-training and post-training. We pooled the Bland-Altman statistics for the experimental and control groups and for pre- and post-training, giving four sets of results. In this study, the mean difference is used as a measure of agreement with the expert consensus. The closer the value of the mean difference to zero, the better the agreement between the expert consensus and the novice. Therefore, we were interested in whether the training intervention could shift this statistic to a value close to zero, i.e. bringing decisions made by novices closer to those made by the experts.

The second type of analysis undertaken was Signal Detection Theory (SDT). SDT predicts accuracy of the fitness-to-drive recommendations made by novices, and detects any change in decision strategy of novices at post-training. Performance measures of SDT are frequently calculated using hit rate and false alarm rate. In relation to this research, the hit rate is described as the probability of correctly classifying a driver as ‘not fit-to-drive’ and the false alarm rate is the probability of incorrectly classifying a driver as ‘not fit-to-drive’. Performance of individual novice participants was evaluated with two measures in this study, the response bias (*c*) and accuracy (*A*_*z*_), the area under the receiver operating curve (ROC). The two measures, *c* and *A*_*z*_, were calculated as described by Stanislaw and Todorov [23]. A change in response bias following training can be interpreted as a positive effect depending on its implications to real fitness-to-drive assessment practices. The area under the ROC curve (ROC area) provides a measure of accuracy, and an increased area under the curve is an indication of a positive effect of training intervention. When response bias *c* equals 0, the novice favours neither response. When *c* is positive, the novice tends to assess scenarios as noise (‘fit-to-drive’). When *c* is negative, the novice tends to assess scenarios as signal (‘not fit-to-drive’), and more likely to underestimate a driver’s capacity for fitness-to-drive (referred to as ‘low-risk’ decision strategy herein).

Available case scenarios were classified into signal and noise scenarios based on the fitness-to-drive recommendations made by expert consensus. To achieve this, the approach used by Harries et al. [25] was adopted. Mean expert recommendation across pre-training scenarios was calculated; mean = 2.15 with 95% confidence interval (CI) (2.006, 2.3). Those scenarios where the experts’ average rating was less than the 95% CI lower bound (2.006) were identified as signal scenarios; those scenarios where experts’ average rating was greater than lower bound of the 95% CI constituted noise scenarios. This approach gave 24 signal scenarios and 23 noise scenarios for pre-training and 7 signal and 10 noise scenarios for post-training.

Data were then analysed as a rating task as outlined by Stanislaw and Todorov [23]. According to SDT, judges (in this case novice occupational therapy driver assessors) in a rating task with r possible outcomes (in the current study *r* = 4) base their decision (fitness-to-drive recommendation) on r-1 criteria; separate criteria is used to distinguish r possible rates/outcomes. For this, an iterative procedure as described by Stanislaw and Todorov [23] was used; this resulted in three pairs of hit and false alarm rates for a novice at each time point. The three pairs of hit and false alarm rates for a time point (pre-training or post-training) were then used to calculate response bias values (*c*_*1*_, *c*_*2*_ and *c*_*3*_) and the area under ROC curve (*A*_*z*_). A two-factor mixed design analysis of variance (ANOVA) was conducted to examine the effect of training on group and time points, and the effect size was calculated.

## Results

**Fig. 1:**
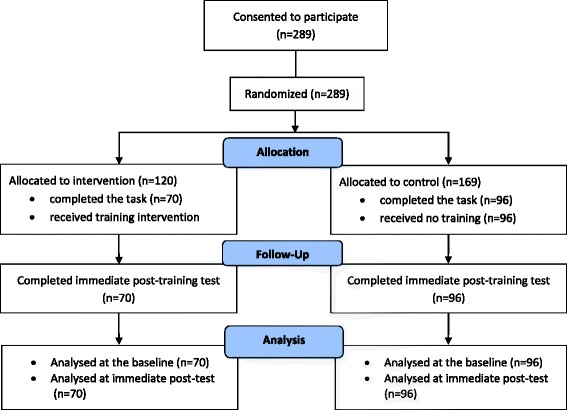
Study Flow Chart (CONSORT Flow Diagram, [26])

Of the 289 participants, randomisation resulted in 120 participants being allocated to the intervention group and 169 to the control (Fig. 1). A total of 166 participants completed the whole task and therefore the data were included for analysis: 70 in the intervention group and 96 in the control. One hundred and forty five of the participants were female (Table 1). Distribution of demographic data between the experimental and control groups were compared using t-tests for age and Mann-Whitney U test for stage of training and driving behaviour. No statistically significant differences were found: t(164) = − 0.73, *p* = 0.46 for age; U = 3320, z = − 0.13, *p* = 0.89 for stage of training; and U = 3344, z = − 0.05, *p* = 0.95 for driving behaviour.

**Table 1:** Participant demographics

Characteristics	Intervention Group (*n* = 70)	Control Group (*n* = 96)
Age in years (mean, SD)	26.1, 7.3	25.2, 7.5
Male	10, 14.3%	11, 11.5%
Female	60, 85.7%	85, 88.5%
Australia	24	39
UK	46	57
Stage		
Final year, % within the group	34, 48.6%	46, 47.9%
Mid stage	22, 31.4%	34, 35.4%
First year	14, 20%	16, 16.7%
Driving behaviour		
Blank	1, 1.4%	3 (3.1%)
Don’t hold a driving licence	4 (5.7%)	11 (11.4%)
Hold a licence; never drive	5 (7.1)	6 (6.3%)
Hold a licence; drive every few months	3 (4.3%)	3 (3.1%)
Hold a licence; drive monthly	1 (1.4%)	2 (2.1%)
Hold a licence; drive weekly	13 (18.6%)	6 (6.3%)
Hold a licence; drive daily	43 (61.4%)	65 (67.7%)

*SD* Standard Deviation

### Mean novice decisions versus mean expert decisions

**Fig. 2:**
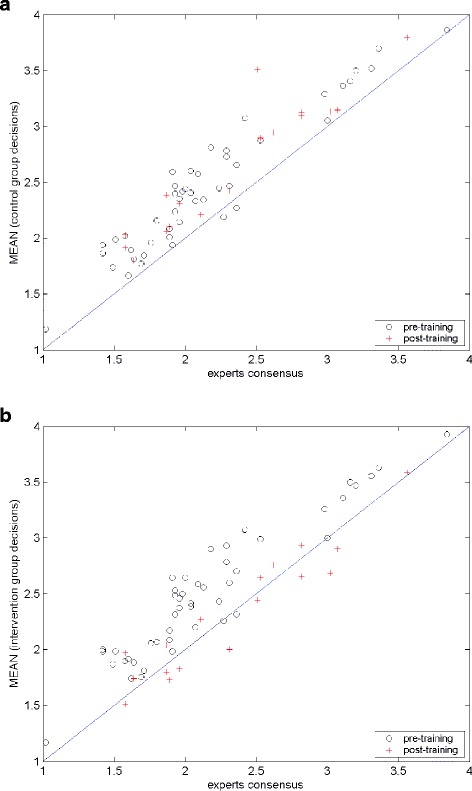
Mean novice decisions versus mean expert decisions: **a**) Control group, **b**) Intervention group

Figure 2 shows the mean score for each scenario, comparing the mean score of the novices plotted against the mean score of the experts. Results from pre-training novices are shown as circles (o) and results from post-training novices as plus sign (+). The solid line along the diagonal is the equality line and represents perfect agreement. This graph enabled us to visually inspect novice consensus fitness-to-drive recommendations in a single graph and see whether the training had any positive effect. Data points for untrained novices, for control group at both pre- and post-training as shown in Fig. 2a, and for the intervention group at the pre-training, as shown in Fig. 2b, are off the equality line. On the other hand, the data points for the trained novices are scattered closer to the equality line, as can be seen in Fig. 2b, for the intervention group at post-training. This indicates that the mean recommendations from trained novices are in stronger agreement with expert consensus than untrained novices. In other words, there was a positive effect of the training on the fitness-to-drive recommendations of novices.

### Agreement between novice and expert recommendations

Agreement between novice and expert consensus fitness-to-drive assessments were calculated using the Bland-Altman test. For each participant, we subtracted the expert consensus decision from each individual participant’s recommendations in a case-by-case manner. For each participant, we estimated the mean and standard deviation (SD) of differences at pre-training and post-training. We then pooled the results from control and intervention groups. Table 2 lists the mean and standard deviations of mean differences for each group. The mean Bland-Altman statistics for the two study groups were very similar at pre-training: 0.30 for the control group and 0.33 for the intervention group. A positive value for the mean indicates a bias towards giving higher rates (i.e. tendency to recommend ‘fit-to-drive’) when compared to experts. At the post training, the control group showed no significant change from pre-training to post-training (t(96) = −.69; *p* = .5), whereas the intervention group exhibited a significant change (t(69) = 6.89; *p* < 0.001). For the intervention group, the mean difference compared with the experts’ recommendations dropped down to 0.02 with 95% CI from −.13 to .09 at post-training. Effect size calculated at the post-training demonstrated a moderate effect (d = .69, *r* = .32) (Cohen, 1988). It can be seen that zero lies within the 95% confidence interval (CI) for the intervention group mean at the post-training. A conventional interpretation of the 95% CI would suggest no bias. However, the negative value of the median (− 0.22) for the intervention group at post training indicates that a significant number of participants shifted their fitness-to-drive recommendation strategy, and gave lower recommendations (i.e. tendency to recommend ‘not fit-to-drive’) than they did before training.

**Table 2:** Comparison of control and training groups of novices, before and after training periods, with experts’ consensus recommendations

	Pre-training period				Post-training period			
	med	Mean (SD)	95% CI for mean		Med	Mean (SD)	95% CI for mean	
			Lower bound	Upper bound			Lower bound	Upper bound
Control	0.28	0.30 (0.36)	0.22	0.37	0.39	0.31 (0.41)	0.22	0.41
Intervention	0.28	0.33 (0.41)	0.24	0.42	−0.22	−0.02 (0.56)	−0.13	0.09

*SD* Standard Deviation, *CI* Confidence Interval

The two-factor ANOVA confirmed that both group and time-point factors had significant main effects and a significant interaction between the two factors. There was a significant interaction between the group and time-point factors: F(1,164) = 50.02, *p* < .01. Partial eta sq. = .234, with a large effect. Simple main effect analysis revealed a significant effect for time-point in the intervention group but not in the control group. In other words, the intervention group were significantly different from the control group after receiving the training. Testing for simple main effects of group factor at both levels of time-point factor revealed that the group factor did not have a significant effect at pre-training (F(1,164) = .29, *p* = .59), but had significant effect at post-training (F(1,164) = 19.41, *p* < .01). Simple main effects of the time-point factor was significant in intervention group (F(1,164) = 78.8, *p* < .01), but not in the control group F(1,164) = .25, *p* = .62). The results demonstrated that the training altered the fitness-to-drive recommendations of the novices who received the intervention.

### Accuracy of novices’ fitness-to-drive recommendations

The accuracy of novices’ fitness-to-drive recommendations for the scenarios was made using Signal Detection Theory. We tested assumptions of normality using Kolmogorov-Simirnov test. Distributions were non-normal in one or other groups for hit rate, false alarm rate, response bias (*c*) and ROC area (*A*_*z*_). Therefore, a Mann-Whitney U test was used to test for the difference between the intervention (trained) and control groups to test significance level of difference between groups. At the post-training stage *c*_*1*_ the intervention group was significantly different from pre-training, while no such change was observed for the control group. The mean value of *c*_*1*_ for the intervention group changed from 1.30 to 0.84 (Table 3). The mean value of *c*_*2*_ also decreased for the intervention group at post-training, hence demonstrating a shift towards a ‘low-risk’ strategy. Therefore the training intervention increased novices’ tendency to recommend ‘not fit-to-drive: suspend license’ instead of ‘not fit-to-drive: rehabilitation to be completed’. When interpreted against ‘real practice’, it could be said that the novices who had been trained were able to detect more cases of ‘not fit-to-drive’ than those who had not received the training.

**Table 3:** Accuracy (*A*_*z*_, area under ROC curve) and bias (*c*_*1*_, *c*_*2*_ and *c*_*3*_)

	Pre- training				Post-training			
	Control		Intervention		Control		Intervention	
	mean	SD	mean	SD	mean	SD	mean	SD
*A*_*z*_	0.78	0.08	0.77	0.07	0.80	0.10	0.81	0.13
*c*_*1*_	1.28	0.50	1.30	0.50	1.34	0.57	0.84	0.86
*c*_*2*_	−0.21	0.65	−0.21	0.73	−0.05	0.78	−0.52	0.96
*c*_*3*_	−1.19	0.48	−1.10	0.50	−1.1	0.56	−1.20	0.67

Results from the Mann-Whitney U test for the accuracy and response bias measures and corresponding effect size measures (Glass rank biserial correlation) are given in Table 4. Across pre-training and post-training (at both time points), the control (*n* = 96) and intervention groups (*n* = 70) did not differ significantly in accuracy (*A*_*z*_) of their decisions (Mann Whitney U = 3129, *p* > 0.05, and the Glass rank biserial correlation r_g_ = < 0.1, showing no effect in Cohen’s (1988) classification, at pre-training; and U = 3620, p > 0.05, and r_g_ = < 0.1, showing no effect, at post-training). At pre-training, the control (*n* = 96) and intervention groups (*n* = 70) did not differ significantly in response bias measures (c_1_, c_2_ and c_3_). On the other hand, an independent samples Mann-Whitney U test showed significant differences in response bias values at post-training: for example, in Table 4, U = 2244, *p* < 0.01 (two-tailed), the Glass rank biserial correlation r_g_ = 0.33, with a medium effect for c_1_.

**Table 4:** Independent samples Mann-Whitney U test (two tailed *p*) and Glass rank biserial correlation r_g_ for accuracy (*A*_*z*_) and bias (*c*_*1*_, *c*_*2*_ and *c*_*3*_) measures

	Pre-training			Post-training		
	U	*p*	r_g_	U	*p*	r_g_
*A*_*z*_	3129	>.05	0.058	3650	>.05	0.087
*c*_*1*_	3454	>.05	0.008	2244	<.05	0.332, medium
*c*_*2*_	3457	>.05	0.005	2248	<.05	0.331, medium
*c*_*3*_	3714	>.05	0.105	2391	<.05	0.288, small

## Discussion

The decision training was effective in demonstrating a medium effect in changing fitness-to-drive recommendations among novices in the experimental group. Before training, the novices tended to under-detect the drivers who were not safe to drive. In other words, they ‘missed’ these cases. Novices who were trained with the on-line training materials then adopted a low risk strategy which was more closely aligned with expert consensus. These novices thought that failing to detect or identify a not fit-to-drive case scenario had more serious consequences than making a recommendation of fit-to-drive for a driver who was potentially not fit-to-drive. Therefore, they were more able to detect more not fit-to-drive case scenarios. This resulted in a negative valued median for the mean differences between recommendations made by trained novices and expert consensus from the Bland-Altman test, and change in response bias values from the signal detection theory. The changes in the mean differences and in response bias values for the trained novices were found to be statistically significant.

Although trained novices did not fully achieve the experts’ capacity of assessing fitness-to-drive, the training influenced novices to detect more ‘not fit-to-drive’ scenarios. Such a change in their fitness-to-driver recommendation strategy may improve safety for road users and help identify unsafe drivers. Therefore, the training can be considered to provide an added benefit for the clients and other road users in terms of reducing risk.

### Implications for practice and service delivery

Novice occupational therapy driver assessors will be able to use the intervention training, as provided on the website, to potentially improve their fitness-to-drive recommendations. They can reflect on the most influential cues used by the experienced occupational therapy driver assessors when determining, in the first instance, whether a client is fit-to-drive or not, and then further consider the client’s medical prognosis when reflecting on whether driver rehabilitation might be beneficial to facilitate a return to driving. The training materials helped the novices shift their decision criteria to be able to detect more not-fit-to-drive cases without decreasing decision accuracy. The decision strategy adopted after training can be thought of an appropriate bias as it improves road safety by detecting more not-fit-to-drive cases. The decision training developed through this research also has the potential to increase the occupational therapy workforce for fitness-to-drive assessments of people with disabilities and older people. Novice occupational therapists can use the website to improve their skill in this difficult area of practice, and gain confidence to work in this field.

### Limitations of the study and future research directions

The accuracy of the novices’ fitness-to-drive recommendations were measured by comparing their decisions with an expert consensus recommendation. Absence of an absolute truth may depress the accuracy levels calculated. However, the fact that the experts remained fairly consistent in their decisions, as evidenced by the high ICC(2,1) = .97 (95% CI .96–.98) in Phase 1 of this research program [14], provides confidence in their expertise. Although this study was fully powered, many novices did drop out, and others in the intervention group might have not studied the training information very carefully nor paid attention when making recommendations due to apathy or desire to complete the task quickly, thus reducing the overall accuracy of the findings.

While this research included a consensus policy derived from expert OTDAs in the UK and Australia, the research should be expanded to include certified driver rehabilitation specialists from North America, and Europe. Further research should also be conducted to test the ecological validity of the expert fitness-to-drive policy developed in this research. Predictions of client fitness-to-drive derived from our research can be tested against real-world fitness-to drive recommendations to determine how well the policy can predict real-world judgments.

## Conclusions

The decision training was shown to be effective in assisting novices to shift their fitness-to-drive recommendations so as to detect more not fit-to-drive cases without decreasing decision accuracy. The decision strategy adopted by the novices after training can be thought of as an appropriate bias as it improves road safety for users by detecting more not fit-to-drive cases. Training novices to make better fitness-to-drive decisions means occupational therapists are more likely to work in this field, where staff shortages have been documented. More OTDAs, making more accurate fitness-to-drive recommendations will potentially lead to a reduction in unsafe drivers, and an increase in quality of life for those drivers who are safely able to commence or resume driving.

## Additional files

Additional file 1: Cues and Cue levels for guiding fitness-to-drive recommendations. (DOCX 19 kb)

Additional file 2: Example of case scenario. (DOCX 15 kb)

## Abbreviations

ANOVA: ANalysis Of VAriance
CI: Confidence Interval
ICC: Intra-class Correlation Coefficient
OTDA: Occupational Therapy Driver Assessor
RCT: Randomised Controlled Trial
ROC: Receiver Operating Curve
SD: Standard Deviation
SDT: Signal Detection Theory
SJT: Social Judgement Theory
