# Supplementary material for: A randomised controlled trial to test the effectiveness of decision training on assessors’ ability to determine optimal fitness-to-drive recommendations for older or disabled drivers
Source: BMC Med Educ. 2018 Feb 13;18:27. doi: 10.1186/s12909-018-1131-4 (PMC5812197; doi:10.1186/s12909-018-1131-4)
Supplement: Supplementary file 1 — Cues and Cue levels for guiding fitness-to-drive recommendations. (DOCX 19 kb) [file 12909_2018_1131_MOESM1_ESM.docx]

# Cues and Cue levels for guiding fitness-to-drive recommendations

| **Cue** | **Description of cue** | **Cue Levels** |
| --- | --- | --- |
| 1. Age | Client’s chronological age. | 1 = 80 years old  2 = 60 years old  3 = 40 years old |
| 2. Driving experience | As well as number of years as a driver, driving experience should also take into account whether the client has had a recent gap in their driving career and why it occurred. | 1 = Client has been driving for less than 3 years  2 = Client has been driving 3-7 years  3 = Client has been driving for more than 7 years |
| 3. Driving history | Driving history can include the number of reported accidents, the types of vehicles driven and weather conditions driven in. Where recall is an issue, a family member has verified this information. | 1 = Client has had a major accident in the last 12-months  2 = Client has had a few minor scrapes in the last 12-months  3 = Client has had no accidents in the last 12-months |
| 4. Current driving needs | A client’s personal driving needs include where they tend to drive (for instance, locally on familiar roads, versus in unfamiliar areas). Driving needs can also refer to how often the client drives and the time of day, as well as if they tend to drive on their own or with others present. | 1 = Client drives predominantly in unfamiliar areas  2 = Client drives predominantly in the local area with only occasional trips to unfamiliar areas  3 = Client drives predominantly in the local / familiar area |
| 5. Physical Skills | Includes the driver’s muscle strength, endurance, tone, grip strength and range of movement, and the driver’s psychomotor reaction time. Vehicle modifications or compensatory strategies may be possible to aid specific physical skills or alleviate symptoms of fatigue and pain. Dependent on the specific issues these could include changing to power assisted steering or using hand controls. | 1 = Physical skills do not support safe driving (no vehicle modifications / compensatory strategies suitable)  2 = Minor problems with physical skills noted, even with vehicle modifications / compensatory strategies made  3 = Physical skills support safe driving |
| 6. Cognitive and / or perceptual skills | Cognitive skills include concentration, memory, planning, and metacognitive ability such as insight into own limitations. Perceptual skills include visuospatial ability. This may be observed through failing to check mirror, not signalling before turning and poor negotiation of intersections / junctions.  Vehicle modifications may be possible to aid specific perceptual skills. Dependent on the specific issues these could include adding a panoramic mirror to help the client to check the mirror more frequently. | 1 = Cognitive and / or perceptual skills do not support safe driving and does not demonstrate capacity for learning and improvement  2 = Minor cognitive and / or perceptual problems identified but demonstrates capacity for learning and improvement  3 = Cognitive and / or perceptual skills support safe driving |
| 7. Sensory functions | Includes tactile sensation, proprioception (awareness of position in space), and vision. | 1 = Sensory functions do not support safe driving  2 = Some sensory problems noted but meets legal requirements  3 = Sensory functions support safe driving |
| 8. Driver behaviour | This refers to psychosocial behaviour. Behaviours of concern include impulsivity, disinhibition, risk taking, aggression, or poor frustration tolerance. | 1 = Behaviour shown does not support safe driving  2 = Some behaviour problems identified  3 = Behaviour shown supports safe driving |
| 9. Road law knowledge and / or road craft | Road law knowledge involves applying road laws whilst driving.  Road craft includes an understanding of the how the car will respond in specific situations (such as knowing that a car may skid if the wheels go from tarmac to gravel at speed). This also requires adjusting driving to match the demands of the situation (for instance, slowing down when driving in a residential area in case pedestrians walk into the road). | 1 = Road law knowledge and / or road craft does not support safe driving  2 = Some problems with road law knowledge and / or road craft identified  3 = Road law knowledge and / or road craft support safe driving |
| 10. Vehicle handling skills | These skills include managing the steering ability, braking speed and car controls such as pedal use. | 1 = Vehicle handling does not support safe driving  2 = Some vehicle handling problems identified  3 = Vehicle handling supports safe driving |
| 11. Driving instructor interventions | Verbal prompts may include a reminder to use the indicator, or to adjust the distance from the car in front.  Physical interventions are as a result of serious driver errors requiring the driving instructor to take control of the vehicle to maintain safety. This could include braking or reaching over to steer the car. | 1 = Driving instructor provides one physical intervention  2 =Driving instructor provides a verbal prompt  3 = No physical or verbal interventions made |
| 12. Medical prognosis | Knowledge about whether the driver’s medical condition is stable or may deteriorate, as may occur with a diagnosis of dementia, Multiple Sclerosis, Huntington’s disease, Parkinson’s Disease and Motor Neurone Disease.  The medical condition has been confirmed by the physician. | 1 = Deterioration expected, likely to impair safe driving  2 = Deterioration not expected but possible  3 = Medically stable |
