# Supplementary material for: A randomised controlled trial to test the effectiveness of decision training on assessors’ ability to determine optimal fitness-to-drive recommendations for older or disabled drivers
Source: BMC Med Educ. 2018 Feb 13;18:27. doi: 10.1186/s12909-018-1131-4 (PMC5812197; doi:10.1186/s12909-018-1131-4)
Supplement: Supplementary file 2 — Example of case scenario. (DOCX 15 kb) [file 12909_2018_1131_MOESM2_ESM.docx]

**Example of case scenario**

The client you are assessing, who is a private car driver, lives on the outskirts of the city / metropolitan area. Their doctor, who would like them to have a comprehensive driver assessment, has referred them to you. They are suitable for on-road assessment as they are medically fit-to-drive, they do not have unilateral spatial neglect (lack of awareness of one side) and they meet the legal vision standards for driving. Initially you undertake a client-based assessment using a battery of tests in the clinic, e.g. range of movements testing, pain questionnaire and medication screen, etc. You decide to proceed to an on-road assessment, which involves you taking the client out, with a driving instructor in a dual-controlled car, in an area not familiar to them. You sit in the back to observe their actual driving behaviour and are going to make your final recommendation from the findings of the comprehensive driver assessment.

The results of the comprehensive assessment are shown below. You can read a description of each cue by using your mouse to hover over the cue label.

| Age | 60 years old |
| --- | --- |
| Driving Experience | Client has been driving for more than 7 years |
| Driving History | Client has had no accidents in the last 12-months |
| Current Driving Needs | Client drives predominantly in the local area with only occasional trips to unfamiliar areas |
| Physical Skills | Physical skills do not support safe driving (no vehicle modifications / compensatory strategies suitable) |
| Cognitive and / or perceptual skills | Minor cognitive and / or perceptual problems identified but demonstrates capacity for learning and improvement |
| Sensory functions | Some sensory problems noted but meets legal requirements |
| Driver behaviour | Behaviour shown does not support safe driving |
| Road law knowledge and / or road craft | Some problems with road law knowledge and / or road craft identified |
| Vehicle handling skills | Vehicle handling supports safe driving |
| Driving instructor interventions | Driving instructor provides a verbal prompt |
| Medical prognosis | Deterioration not expected but possible |

What is your fitness-to-drive recommendation for this client?

- Fit-to-drive – unrestricted licence
- Fit-to-drive – with conditions, for example, using an automatic car
- Not fit-to-drive – Driver rehabilitation to be completed
- Not fit-to-drive –Suspend or cancel licence
